# Dehydroepiandrosterone (DHEA) Feeding Protects Liver Steatosis in Obese Breast Cancer Rat Model

**DOI:** 10.3390/scipharm85010013

**Published:** 2017-03-20

**Authors:** Reza Hakkak, Andrea Bell, Soheila Korourian

**Affiliations:** 1 Department of Dietetics and Nutrition, 4301 W. Markham St., Mail Slot 627, Little Rock, AR 72205, USA; BellAC@archildrens.org; 2 Pediatrics, University of Arkansas for Medical Sciences, 4301 W. Markham St., Little Rock, AR 72205, USA; 3 Arkansas Children’s Research Institute, 13 Children’s Way, Little Rock, AR 72202, USA; 4 Department of Pathology, University of Arkansas for Medical Sciences, W. Markham St., Little Rock, AR 72205, USA; Korouriansoheila@uams.edu

**Keywords:** DHEA, obesity, liver steatosis, weight loss

## Abstract

Obesity is a major health problem in the US and globally. Obesity is associated with the risk of cardiovascular disease, type 2 diabetes, cancers, hyperlipidemia, and liver steatosis development. Dehydroepiandrosterone (DHEA) is a dietary supplement used as an anti-obesity supplement. Previously, we reported that DHEA feeding protects 7,12-dimethylbenz(a)anthracene (DMBA)-induced mammary tumors. The objectives of this study were to investigate the effects of obesity and DHEA feeding on liver steatosis, body weight gain, and serum DHEA, DHEA sulfate (DHEA-S), insulin-like growth factor-1 (IGF-1), and insulin-like growth factor binding protein-3 (IGFBP-3) levels. Female Zucker rats were randomly assigned to either a control diet or a control diet with DHEA supplementation for 155 days. Livers were collected for histological examination. Serum was collected to measure DHEA, DHEA-S, IGF-1, and IGFBP-3. Our results show that DHEA-fed rats had significantly less liver steatosis (*p* < 0.001) than control-fed rats and gained less weight (*p* < 0.001). DHEA feeding caused significant decreases (*p* < 0.001) in the serum levels of IGF-1 and IGFBP-3 and significantly increased (*p* < 0.001) serum levels of DHEA and DHEA-S. Our results suggest that DHEA feeding can protect against liver steatosis by reducing body weight gain and modulating serum IGF-1 and IGFBP-3 levels in an obese breast cancer rat model.

## 1. Introduction

More than two-thirds (68.5%) of American adults are either overweight or obese [1]. In the US, 34.9% are obese, and 6.4% are extremely obese (grade 3 obesity) [1]. Globally, more than 1.9 billion adults are overweight, and over 600 million adults are obese [2]. Obesity is associated with serious health problems such as cardiovascular disease, type 2 diabetes, certain types of cancers, hyperlipidemia, and liver steatosis [3].

Dehydroepiandrosterone (DHEA) is a dietary supplement that is available in many health food stores. It has been shown that DHEA has potential as an anti-cancer agent and anti-obesity supplement [4]. Epidemiological studies investigating the relationship between DHEA and abdominal fat have conflicting results. In some studies, levels of serum DHEA or the DHEA metabolite DHEA sulfate (DHEA-S) were significantly inversely related to abdominal obesity [5,6]. In contrast, studies in postmenopausal women reported that DHEA-S did not protect against obesity [7]. In laboratory animals (rats and mice), DHEA feeding reduced fat accumulation in both genetic- and diet-induced obesity, and had protective effects against insulin resistance induced by a high-fat diet [8,9].

Fatty liver disease can range from fatty liver alone (liver steatosis) to fatty liver associated with inflammation (steatohepatitis). Fatty liver may occur with the use of alcohol (alcohol-related fatty liver) or in the absence of alcohol (nonalcoholic fatty liver disease). Nonalcoholic fatty liver disease (NAFLD) is a common form of liver disease with a prevalence of up to 34% in the US adult population [10]. Patients with NAFLD can develop progressive liver disease, leading to end-stage liver disease and hepatocellular carcinoma [11]. Ong et al. have shown that patients with NAFLD had a higher mortality rate compared to the general population, and liver disease was a significant cause of death among NAFLD patients [12].

NAFLD is associated with obesity, type 2 diabetes mellitus (T2DM), and hyperlipidemia [13]. During obesity, fat cells become hypertrophic; this hypertrophy has been linked to the dysregulation of adipose tissue-derived chemokines, cytokines, and adipokines, leading to inflammation and contributing to the development of insulin resistance (the precursor to T2DM) [13]. In an insulin-resistant state, insulin is unable to adequately downregulate lipolysis; thus, free fatty acids are released from adipose tissue into circulation (contributing to hyperlipidemia) and transported to the liver [13]. An excess flux of fatty acids from circulation into the liver increases hepatic lipid storage and leads to the development of hepatic steatosis [13].

High serum levels of insulin-like growth factor-1 (IGF-1) and low levels of insulin-like growth factor binding protein-3 (IGFBP-3) are associated with obesity [14,15,16], and obesity is associated with NALFD development [13]. Insulin-like growth factor-1 is a hormone produced predominantly in the liver [17]. When liver function is compromised (such as during NAFLD), serum levels of IGF-1 decline [17]. The relationship between IGF-1 and NALFD is still under investigation to determine if low IGF-1 contributes to the development of NAFLD or is a side-effect of NAFLD [17,18].

Previously, we have shown that DHEA treatment can protect against 7,12-dimethylbenz(a)anthracene (DMBA)-induced breast cancer development [19], but the effects of DHEA on liver steatosis in an obese breast cancer model are unknown. We hypothesized that obesity will increase liver steatosis and DHEA treatment will protect against liver steatosis by reducing body weight gain and modulating serum IGF-1 and IGFBP-3 levels in breast cancer model.

## 2. Results

### 2.1. Body and Liver Weights

**Figure 1:**
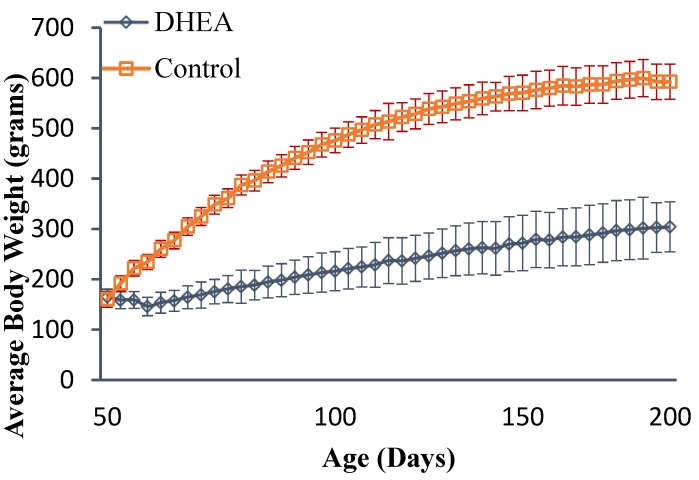
Mean ± SD body weight of obese rats with and without DHEA treatment. (Modified from *Oncol Rep*. **2010**, *24*, 357–362) [19].

All rats gained weight during this experiment. The (mean ± standard deviation (SD)) body weights at several points are shown in Figure 1. The mean ± SD for the final body weight for controls was 593 ± 49 g, and for the DHEA-fed rats was 304 ± 35 g. The DHEA-fed rats gained significantly less weight (*p* < 0.001) than control-fed rats. Table 1 shows the liver weights, expressed as absolute and as percent of body weight. DHEA-fed rats had significantly lower absolute liver weights than control rats (*p* < 0.001). However, when liver weight was expressed as percent of body weight, DHEA-fed rats had significantly higher liver weight than control rats (*p* < 0.001).

### 2.2. Liver Pathology

**Figure 2:**
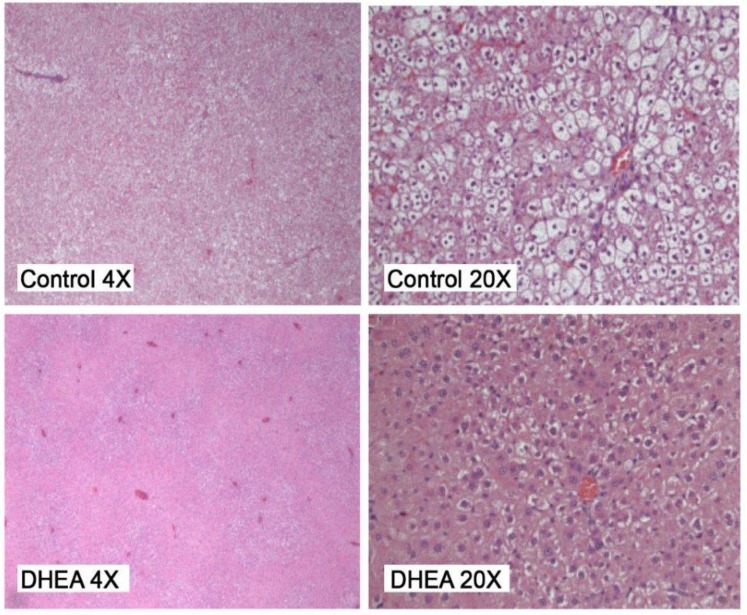
Liver steatosis: obese control shows marked steatosis, original magnification 4× and 20×. Obese DHEA shows less steatosis, original magnification 4× and 20×.

Figure 2 shows photomicrographs of the hepatic parenchyma for control and DHEA-fed rats. Table 1 shows the average liver steatosis score (mean ± standard error). The DHEA-fed rats had a significantly lower (*p* < 0.001) liver steatosis score (1.36 ± 0.44) than control rats (3.21 ± 1.28).

### 2.3. Serum Measurements for IGF-1, IGFBP-3, DHEA, and DHEA-S

DHEA-fed rats had lower (*p* < 0.001) serum IGF-1 and IGFBP-3 than control rats (Table 2). DHEA-fed rats also had higher (*p* < 0.001) serum DHEA and DHEA-S than control rats.

## 3. Discussion

Obesity can increase the risk of breast cancer and liver steatosis development [13,14]. Previously, we reported that DHEA treatment can protect against mammary tumor development [19]. We reported that while only 55% of the obese control rats developed mammary tumors, there were no mammary tumors found in the DHEA-fed rats (*p* < 0.001). Eighteen mammary tumors were detected in the control group, and were classified as 1 benign (6%), 2 intraductal proliferation (IDP) (11%), 11 ductal carcinoma in situ (DCIS) (61%), and 4 invasive ductal and lobular carcinoma (IDC) (22%).

The goals of this study were to use the obese breast cancer model to investigate (1) the long-term effects of obesity and DHEA treatment on body weight gain and liver steatosis and (2) the effects of long-term obesity and DHEA on serum IGF-1, IGFBP-3, DHEA, and DHEA-S.

The current results are additional new data from a previously reported study [19]. In the present study, we found that DHEA-fed rats had significantly lower liver weights and steatosis scores than control rats (*p* < 0.001). DHEA-fed rats also gained significantly less weight (*p* < 0.001) than control rats. DHEA feeding caused lower (*p* < 0.001) serum IGF-1 and IGFBP-3 compared to control rats and higher (*p* < 0.001) serum DHEA and DHEA-S. Our results suggest the important role of lower weight gain in liver steatosis development. This observation suggests that gaining less body weight by DHEA treatment may be responsible for lower liver steatosis [20]. Some studies reported that DHEA can decrease body weight gain [8,21,22].

It has been shown that DHEA can decrease liver steatosis because it is a noncompetitive inhibitor of glucose-6-phosphate dehydrogenase (G6PD). DHEA can decrease liver and adipose tissue G6PD and fatty acid synthase activities, can decrease serum insulin and cholesterol as well as decrease hepatic acyl-CoA cholesterol-acyl transferase activity, and can reduce fat accumulation in both genetic- and diet-induced obesity models, which can have protective effects against insulin resistance induced by a high-fat diet [8].

Abadie et al. [21] investigated the effects of DHEA feeding on serum, adipose, and hepatic tissue fatty acid (FA) profiles and serum free fatty acid (FFA) levels in lean and obese rats. They reported that DHEA treatment lowered FFA levels (*p* < 0.05) in both lean and obese rats. They also reported several significant differences in the FA profile of serum, hepatic, and adipose lipid components. They observed that DHEA-related changes were only in the serum phospholipid (PL), liver PL, and triglyceride fractions. They found a slight but significant decrease in serum FFA levels that may reflect changes in serum PLFA profiles [21]. They suggested that these changes may be related to the DHEA-induced changes in hepatic stearoyl-CoA desaturase, as previously reported [23].

We investigated the effects of different diet (especially soy protein diet) on liver steatosis. Results from our laboratory reported that obesity increases liver steatosis and diet containing soy protein reduced liver steatosis in a breast cancer model [21]. We have also shown that short- and long-term soy protein consumption can decrease liver steatosis in male obese rats [24].

To further understand the effects of DHEA on liver steatosis, we examined DHEA’s effects on key liver steatosis serum markers. Elevated serum IGF-1 and lower IGFBP-3 levels are predictive of the presence of NAFLD. Our laboratory and others have shown that obesity increases the serum IGF-1 and lowers IGFBP-3 levels [14,15,16]. In the present study, we have shown that long-term DHEA feeding reduced both serum IGF-1 and IGFBP-3 levels, which potentially can be effective in reducing liver steatosis. In agreement with our data, Barrett et al. have shown that DHEA treatment reduced body weight gain, adipose tissue weights, and serum insulin levels [7]. We are aware of only one study which used five normal (not obese) men to investigate the effects of short-term DHEA supplementation (1600 mg/day for 28 days) on body fat mass, serum lipid levels, and tissue sensitivity to insulin. They reported that normal men given DHEA had reduced body fat, increased muscle mass, and reduced serum low density lipoprotein cholesterol levels, but tissue sensitivity to insulin was unaffected by short term DHEA administration [25].

It has been reported that DHEA treatment reduces adipose tissue accumulation, induces glucose uptake in adipocytes, and protects against insulin resistance in rats [26,27]. Furthermore, DHEA treatment using obese rats induces anti-obesity effects by decreasing body weight, fat intake, size of specific adipose tissue, and fat accumulation in the liver in obese breast cancer model. DHEA treatment can also alter serum fatty acid profiles and free fatty acid levels, which may contribute to DHEA’s protection against fatty liver [21].

## 4. Materials and Methods

### 4.1. Experimental Design

The animal protocol was approved by the Institutional Animal Care and Use Committee (IACUC) at the University of Arkansas for Medical Sciences (ethical approval code #2851). As previously reported, 43 six-week-old obese (*fa/fa*) female Zucker rats were used [19]. After one week of acclimation, rats were randomly assigned and had ad libitum access to water and a pellet diet of either chow (2016) (Teklad, Madison, WI, USA) as a control diet or pellet chow with the addition of DHEA (Sigma Chemical Co., St. Louis, MO, USA) at a concentration of 6 g of DHEA/kg of chow as a DHEA diet. DHEA is widely used as a food supplement and is considered to be relatively safe. The chow (2016) diet contained corn, corn gluten meal, wheat, wheat middling, soybean oil, and minimum isoflavones. There are several animal studies that used a similar DHEA dose (6 g/kg of diet or 0.6%) to investigate the effects of feeding DHEA on several metabolic changes including weight loss using the Zucker rat model [8,21]. However, there is one study that used regular mice and not obese Zucker rats, and the study reported that mice fed with pellets containing 0.6% DHEA for 3 months showed a significant neuronal loss in the cerebral cortex and hippocampus, a slightly decreased dopamine/dihydroxyphenylacetic acid ratio, and motor impairment [28]. However, there are no reports of toxicity when using 6 g/kg of diet in the obese Zucker rat model.

Rats were housed one per cage and weighed twice per week. All rats were orally gavaged at age 50 days with 65 mg DMBA/kg body weight and were sacrificed 155 days post-DMBA treatment as previously reported [19]. Liver samples were collected for liver histology. Serum was collected to measure DHEA, DHEA-S, IGF-1, and IGFBP-3 levels.

### 4.2. Liver Histology

A board-certified anatomic pathologist evaluated livers in a blinded protocol. Two 3-mm sections of each liver lobe were fixed in 10% buffered formalin for histological examination. Liver sections were fixed in 5% buffered formalin, cut, and stained with hematoxylin and eosin (H&E). Liver sections were evaluated for the presence of microvesicular and macrovesicular steatosis. The percentage of liver cells showing fat accumulation was estimated. A score of 1 to 4 was given to each section, reflecting the relative degree of steatosis in hepatocytes: 1 (<25%), 2 (25%–50%), 3 (51%–75%), and 4 (>75%) [29,30].

### 4.3. Serum Measurements

Serum IGF-1 and IGFBP-3 were measured by using an enzyme-linked immunosorbent assay (ELISA) Kit (IDS, Fountain Hills, AZ, USA). Serum DHEA and DHEA-S were also measured using an ELISA Kit (DSL, Webster, TX, USA).

### 4.4. Statistical Analyses

Statistical analyses were performed as previously reported [19,22,23,24]. Analysis of variance (ANOVA) followed by Student’s *t*-tests were carried out, and *p*-values were reported. Statistical significance was set at *p* < 0.05 (two sided) for individual test and Bonferroni-adjustment applied in significance level for multiple comparisons. We used SAS software (version 9.2; SAS Institute, Inc., Cary, NC, USA) for data analyses.

## 5. Conclusions

Previously, we showed that DHEA-feeding can protect breast cancer development. Our current results suggest that long-term DHEA feeding can also protect against liver steatosis by reducing body weight gain and reducing serum IGF-1 and IGFBP-3 in an obese breast cancer model.

## Figures and Tables

**Table 1:** Body weight (BW, g), liver weight (absolute and as % of body weight), and steatosis score (mean ± SD).

Groups	Body Wt (g)	Liver Wt (g) Absolute	Liver Wt % of BW	Steatosis Score
Control	593 ± 49	21.78 ± 2.88	3.928 ± 0.61	3.21 ± 1.28
DHEA	304 ± 35 ^a^	18.17 ± 2.55 ^a^	6.57 ± 0.74 ^a^	1.36 ± 0.44 ^a^

^a^ Mean is significantly different from control, *p* < 0.001. DHEA: dehydroepiandrosterone; Wt: weight.

**Table 2:** Effects of DHEA supplementation on serum DHEA, DHEA-S, IGF-1, and IGFBP-3 (mean ± SE).

Groups	DHEA	DHEA-S	IGF-1	IGFBP-3
Control	3.55 ± 0.38	0.16 ± 0.01	1,295.82 ± 16.65	217.41 ± 2.74
DHEA	748.40 ± 15.15 ^a^	30.80 ± 0.55 ^a^	771.45 ± 7.83 ^a^	168.58 ± 1.73 ^a^

^a^ Mean is significantly different from control, *p* < 0.001. DHEA-S: Dehydroepiandrosterone sulfate; IGF-1: insulin-like growth factor-1; IGFBP-3: insulin-like growth factor binding protein-3.
